# Activating KIR and HLA Bw4 Ligands Are Associated to Decreased Susceptibility to Pemphigus Foliaceus, an Autoimmune Blistering Skin Disease

**DOI:** 10.1371/journal.pone.0039991

**Published:** 2012-07-02

**Authors:** Danillo G. Augusto, Sara C. Lobo-Alves, Marcia F. Melo, Noemi F. Pereira, Maria Luiza Petzl-Erler

**Affiliations:** 1 Laboratório de Genética Molecular Humana, Departamento de Genética, Universidade Federal do Paraná, Curitiba, Brazil; 2 Laboratório de Imunogenética, Hospital de Clínicas, Universidade Federal do Paraná, Curitiba, Brazil; Centre de Recherche Public de la Santé (CRP-Santé), Luxembourg

## Abstract

The *KIR* genes and their HLA class I ligands have thus far not been investigated in pemphigus foliaceus (PF) and related autoimmune diseases, such as pemphigus vulgaris. We genotyped 233 patients and 204 controls for *KIR* by PCR-SSP. *HLA* typing was performed by LABType SSO reagent kits. We estimated the odds ratio, 95% confidence interval and performed logistic regression analyses to test the hypothesis that KIR genes and their known ligands influence susceptibility to PF. We found significant negative association between activating genes and PF. The activating *KIR* genes may have an overlapping effect in the PF susceptibility and the presence of more than three activating genes was protective (OR = 0.49, p = 0.003). A strong protective association was found for higher ratios activating/inhibitory *KIR* (OR = 0.44, p = 0.001). *KIR3DS1* and HLA-Bw4 were negatively associated to PF either isolated or combined, but higher significance was found for the presence of both together (OR = 0.34, p<10^−3^) suggesting that the activating function is the major factor to interfere in the PF pathogenesis. HLA-Bw4 (80I and 80T) was decreased in patients. There is evidence that HLA-Bw4(80T) may also be important as KIR3DS1 ligand, being the association of this pair (OR = 0.07, p = 0.001) stronger than KIR3DS1-Bw4(80I) (OR = 0.31, p = 0.002). Higher levels of activating KIR signals appeared protective to PF. The activating *KIR* genes have been commonly reported to increase the risk for autoimmunity, but particularities of endemic PF, like the well documented influence the environmental exposure in the pathogenesis of this disease, may be the reason why activated NK cells probably protect against pemphigus foliaceus.

## Introduction

Pemphigus is a group of blistering autoimmune diseases of the skin that can be subdivided in two major clinical types: pemphigus foliaceus (PF) and pemphigus vulgaris (PV). The target of the autoantibodies differs between these two subtypes: while in PF the antibodies are directed to desmoglein 1 (dsg1), in PV they are direct to desmoglein 3 or to both desmoglein 1 and desmoglein 3 [1]. Endemic in Brazil, PF was first reported in this country in 1903, when it was thought to be a variant of a superficial mycosis [2]. PF represents a health problem in Brazil with an incidence of 25–35 million/cases/year. The highest prevalence of 3.4% was reported for the Terena Amerindian population that sums a total of about 1,200 individuals, in southern Mato Grosso do Sul State [3]. This disease manifests usually in rural workers and other low-wage earners who live near rivers and are exposed to poor living conditions [4]. The anti-dsg1 antibodies in PF (mainly of the IgG4 subclass) recognize epitopes of the amino-terminal domains of the extracellular portion of desmoglein 1 [5], affecting the intercellular junction. The epidermal cell detachment (acantholysis) produces blistering lesions that may appear as localized or generalized forms, being a suffering and painful disease.

The etiology of PF is still unclear. As a multifactorial disease, there are environmental and genetic factors. The first genetic association with PF was reported in 1989 by our group [6] and was corroborated by further studies [7] that showed the importance of the *HLA-DR* and *HLA-DQ* genes in the susceptibility to PF. The polymorphism of other genes, as those coding for cytokines [8], desmoglein 1 [9], *CTLA4* [10], [11] *CD40L*, *CD40*, *BLYS* and *CD19* [12] has also been analyzed.

NK cells are important for the immune response, and are controlled by activating and inhibitory receptors. The killer-cell immunoglobulin-like receptors (KIR) and their cognate HLA class I ligands are crucial for NK regulation [13]. The ligands of several inhibitory (2DL and 3DL) and activating (2DS and 3DS) KIR have been described. The KIR2DL1 recognizes HLA-C2 while KIR2DL2 and 2DL3 recognize the allotypes of HLA-C named C1 and a few C2 allotypes [14]. KIR2DS1 exhibits weak recognition for HLA-C2 and KIR2DS2 appears not to recognize any HLA epitope [15], [16]. KIR3DL1 binds the epitope Bw4, that comprises about 40% of the HLA-B allotypes and some of the HLA-A molecules (A*23, A*24, A*25 and A*32) [17]. The Bw4 positive HLA-A molecules and also a subset of the HLA-B Bw4 molecules that have isoleucine at position 80 (Bw4-80I) showed stronger interaction with KIR receptors than Bw4-80T (threonine) [18], [19]. Despite HLA-B epitopes are currently regarded as the most important KIR3DL1 ligands, we recently suggested that both HLA-A and HLA-B allotypes may be equally important for NK cell function [20]. Moreover, it has been suggested that KIR3DS1 also interacts with Bw4 molecules [21], even with no direct evidence.

Natural killer (NK) cells may play an important role in autoimmune diseases of the skin [22]. Takahashi *et al.* [23] showed that a higher percentage and number of NK cells are present in the peripheral blood of PV patients. Moreover, the NK cell of patients exhibited lower levels of perforin and granzime. Evidences that NK may play a role in the pathobiology of PV were also reported [24]. *KIR* polymorphism and combinations KIR/HLA have been associated with several diseases, including autoimmune [25], but the importance of NK and *KIR* in pemphigus foliaceus has not yet been explored. The aim of this work was to verify if *KIR* polymorphism and their known HLA ligands influence the susceptibility or resistance to PF.

## Results

The frequencies of all the KIR genes analyzed in patients and controls are listed in Table S1. The absence/presence polymorphism of *KIR2DS1*, *KIR2DS3*, *KIR3DS1* and *KIR2DL5* was found negatively associated with PF, while *KIR3DL1* was positively associated to the disease in Euro-descendants (Table 1). As extensively known, *KIR* haplotypes are subdivided in two major haplogroups (A and B), being the A haplogroup characterized by the presence of only *KIR2DS4* as classic activating gene [26]. We found that the A/A genotype is associated to a higher susceptibility to PF and that the presence of B haplotypes is protective (Table 2).

**Table 1:** Genetic association between pemphigus foliaceus and five *KIR* genes.

Gene		Patients		Controls				
		n	F (%)	n	F(%)	P	OR	CI
2DS1	Euro	45 (143)	31.5	65 (131)	49.6	0.002	0.46	0.28–0.76
	Afro	35 (90)	38.9	31 (72)	43.1	0.590	0.84	0.44–1.58
2DS3	Euro	27 (144)	18.8	44 (130)	33.8	0.004	0.45	0.26–0.78
	Afro	30 (89)	33.7	20 (73)	27.4	0.386	1.34	0.68–2.65
3DS1	Euro	42 (142)	29.6	58 (131)	44.3	0.011	0.54	0.33–0.89
	Afro	36 (89)	40.4	28 (73)	38.4	0.791	1.09	0.58–2.06
2DL5	Euro	65 (144)	45.1	81 (130)	62.3	0.004	0.49	0.30–0.87
	Afro	55 (89)	61.8	41 (75)	54.7	0.356	1.34	0.72–2.50
3DL1*	Euro	135 (144)	93.8	116 (131)	88.6	0.126	1.93	0.81–4.60
	Afro	84 (89)	94.4	64 (73)	87.7	0.130	2.35	0.75–7.39

In parentheses, the total number of individuals in the sample. Only genes which presented significant (P<0.05) association in at least one population sample are shown. The frequencies of all *KIR* genes are presented in Table S1.

Euro: predominantly European background; Afro: mixed subsample, with predominantly African background.

* For the total sample (combining the Euro and Afro populations) we found a significant association for *KIR3DL1*: OR  = 2.1, 95% CI 1.05–4.14, p = 0.033.

**Table 2:** The A/A genotype is associated to increased susceptibility to pemphigus foliaceus.

Genotypes		Patients		Controls				
		*n*	*F (%)*	*n*	*F(%)*	*P*	*OR*	*95% CI*
A/A	Euro	50 (145)	34.5	27 (134)	20.1	0.007	2.08	1.21–3.60
	Afro	25 (90)	27.8	18 (72)	25.0	0.689	1.15	0.57–2.34
B/x	Euro	95 (145)	65.5	107 (134)	79.9	0.007	0.48	0.28–0.83
	Afro	65 (90)	72.2	54 (72)	75.0	0.689	0.87	0.43–1.75

In parentheses, the total number of individuals in the sample. B/x indicates A/B or B/B genotypes. Euro: predominantly European background; Afro: mixed subsample, with predominantly African background; Total: total sample.

**Figure 1:**
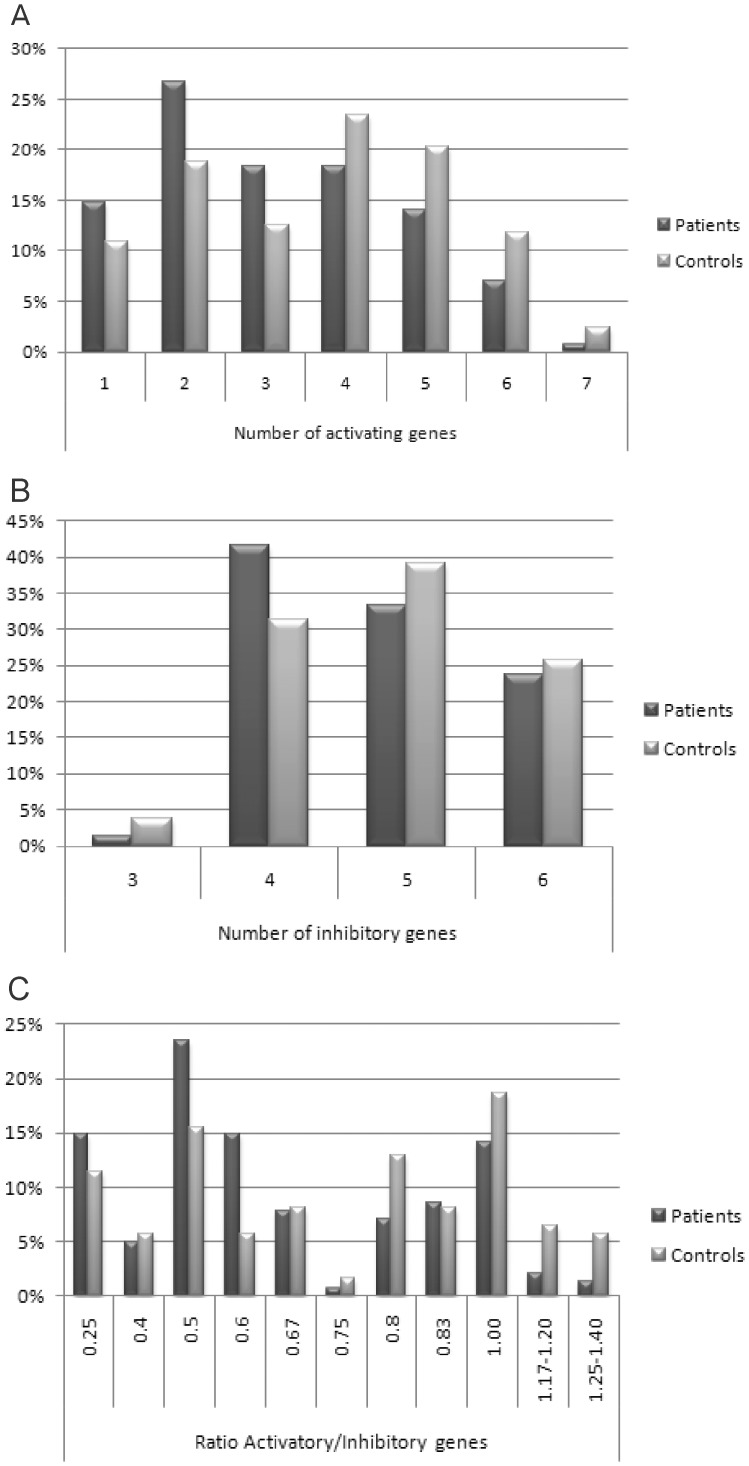
Frequency of the number of activating and inhibitory *KIR* genes in patients and controls. Only the Euro population was considered. (**A**) *KIR2DL4* was counted as activating gene and the deleted *KIR2DS4* was not counted as activating or inhibitory. Significant negative association was found for the presence of more than 3 activating genes (OR  = 0.49; 95% CI = 0.30–0.79; p = 0.003). (**B**) *KIR3DL3* was not included as inhibitory. (**C**) Significant negative association was found for the ratio higher than 0.75 (OR  = 0.44; 95% CI  = 0.27–0.73; p = 0.001).

The number of inhibitory and of activating genes was compared between patients and controls. The function of *KIR2DL4* is controversial, since it has an inhibitory motif (ITIM) in the intracellular domain [27] and an arginine in the transmembrane domain, putatively responsible for activating function [28]. It has been reported that the predominant function of *KIR2DL4* is activating [28], [29] and for that reason, this gene was considered as activating in our analyses. No protein product or ligand was reported for the *KIR3DL3* gene thus far [30]. Therefore, this gene was removed from our analyses, considering that its function is still obscure. The deleted form of *KIR2DS4* was counted neither as activating nor as inhibitory. We found a significant association for the presence of more than three activating genes (OR  = 0.49, 95% CI  = 0.30–0.79, p = 0.003), which also means an odds ratio of 2.08 (95% CI  = 1.25–3.32) for the presence of three or less activating genes (Figure 1A). For inhibitory genes, no statistical differences were found (Figure 1B). Further, we analyzed the ratio activating/inhibitory genes; the frequencies are shown in Figure 1C. A strong negative association was found for the ratio equal or higher than 0.75 (OR  = 0.44; 95% CI  = 0.27–0.73; p = 0.001).

The model tested in regression analyzes was performed only in the Euro descendants sample and it included the follow components: Presence of each of the negatively associated genes (*KIR2DS1, KIR2DS3, KIR3DS1* and *KIR2DL5*), presence of HLA ligands (Bw4, C1 and C2), the presence of A/A or B/x haplotypes, the presence of more than three activating genes and the occurrence of activating/inhibitory genes ratio equal or higher than 0.75. The logistic regression analysis showed that the presence of more than three activating genes alone explained the result (OR  = 0.25, 95% CI  = 0.13–0.49, p<10^−3^, log likelihood  = 195.502).

The frequent deletion of 22 pb in a group of *KIR2DS4* alleles reported by Hsu *et al.* [*31*] generates a truncated protein with no transmembrane and cytoplasmic domains not expressed on the cell surface. We investigated if this deletion, and consequently the loss of the *KIR2DS4* activating function, interferes in PF susceptibility, and no significant result was found (p>0.05).

We analyzed all known HLA class I KIR ligands (Table 3). The presence of one or more Bw4 epitopes (Bw4 80T and Bw4 80I from the *HLA-A* or *HLA-B* loci) was negatively associated (OR  = 0.45, p = 0.005). Bw4 80T was also associated to protection against the disease (OR  = 0.37, p = 0.001) but we did not find any association for Bw4 80I individually. We observed a strong and positive association for the presence of Bw6 (OR  = 4.28, p<10^−3^). We also analyzed the association between individuals who present only Bw4 epitopes, individuals carrying both Bw4 and Bw6 and individuals carrying only Bw6. For these analyses, we considered both HLA-A and HLA-B loci. The effect was compatible with the absence of dominance and epistasis (only Bw4: OR = 0.24, p<10^−3^; both Bw4 and Bw6: OR  = 0.61, p = 0.05; only Bw6: OR  = 1.92, p = 0.03). Next, we tested if the Bw4 epitopes were in linkage disequilibrium (LD) with HLA class II alleles associated to PF reported by Pavoni et al. (2003). In that study, the authors separated the *HLA-DRB1* alleles in three groups: susceptibility, neutral and protective. We found that LD between the susceptibility group of *HLA-DRB1* alleles and Bw4(80T) is negative (D’  = −1.00, p = 0.001). Bw4(80I) was also in negative LD with this group of HLA alleles (D’  = −0.47, p = 0.001) while LD with Bw6 was positive (D’  = 0.59, r^2^ = 0.13 and p<10^−3^).

**Table 3:** Association analyzes between pemphigus foliaceus and HLA ligands of KIR.

KIR Ligands		Patients		Controls				
		*n*	*f (%)*	*n*	*F(%)*	*P*	*OR*	*95% CI*
Bw4*	Euro	64 (99)	64.6	56 (71)	78.9	0.045	0.49	0.24–0.99
	Afro	42 (64)	65.6	35 (42)	83.3	0.045	0.38	0.15–0.99
	Total	106 (163)	65.0	91 (113)	80.5	0.005	0.45	0.26–0.79
Bw4 80I	Euro	57 (99)	57.6	47 (72)	65.3	0.307	0.72	0.38–1.35
	Afro	38 (64)	59.4	30 (42)	71.4	0.210	0.58	0.25–1.34
	Total	95 (163)	58.3	77 (114)	67.5	0.110	0.45	0.41–1.11
Bw4 80T	Euro	15(98)	15.3	16 (66)	24.2	0.144	0.56	0.25–1.22
	Afro	5 (61)	8.2	14 (41)	34.2	0.001	0.17	0.06–0.53
	Total	20 (159)	12.6	30 (107)	28.0	0.001	0.37	0.20–0.69
Bw6	Euro	91 (96)	94.8	57 (66)	86.4	0.060	2.87	0.92–9.01
	Afro	58 (61)	95.1	30 (41)	73.2	0.001	7.09	1.84–27.04
	Total	149 (157)	94.9	87 (107)	81.3	<10^−3^	4.28	1.81–10.13

In parentheses, the total number of individual in the sample. Euro: predominantly European background; Afro: mixed subsample, with predominantly African background; Total: total sample. *at least one Bw4 epitope (80I from *HLA-A* or *HLA-B* loci or 80T).

**Figure 2:**
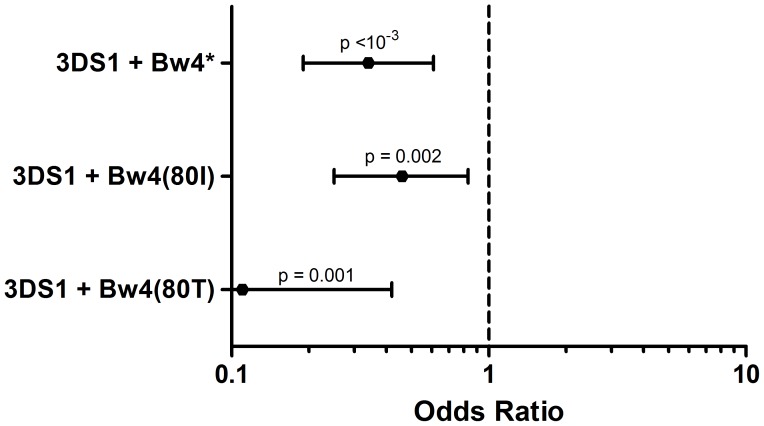
Association analyzes for the combinations KIR x HLA ligands. The bars indicate the 95% confidence interval and the black spot indicates the OR. The results for the Euro sample are shown. The results for the two population strata and the complete information about *KIR* and *HLA* combinations are found in Table S2. *at least one Bw4 epitope (80I from *HLA-A* or *HLA-B* loci or 80T).

The KIR x HLA combinations were analyzed and are shown in Figure 2 and Table S2. We found negative associations for combinations including *KIR3DL1*/*S1* and Bw4 ligands. We did not find any association for *KIR2DL1*, *KIR2DL2*/*3* and *KIR2DS1* combined with their HLA-C ligands (data not shown).

## Discussion

In this study we describe the *KIR* gene frequencies in an urban population living in Western Brazil (Table S1) and, for the first time, we show a genetic association between susceptibility to pemphigus foliaceus and *KIR* genes and their HLA ligands.

All negatively associated genes (*KIR2DS1*, *KIR2DS3*, *KIR3DS1* and *KIR2DL5*– Table 1) are not present in haplotype A, what could indicate, at first moment, that the presence of the B haplotype and not of each isolated gene, may protect against PF. In fact, we found a genetic association for the presence of B haplotypes (Table 2). In addition, we analyzed all pairs of associated genes for the presence of both, presence of one or the other and the absence of both (data not shown). Based on this, we hypothesized that the activating *KIR* could exhibit an additive effect for PF protection. Considering that combinations of several activating genes are typically found in B haplotypes [26], we hypothesized that the KIR activating function, rather than the isolated receptors, could be the cause of the differential susceptibility. Counting the number of activating genes in each individual, it became clear that the presence of a higher number of activating genes has a protective effect (Figure 1A). As expected by this hypothesis, the number of inhibitory genes did not differ significantly between patients and controls (Figure 1B). It is consensus that the balance of activating and inhibitory signals has a major effect on NK cell function [32]. Bearing that in mind, we evaluated the ratio activating/inhibitory genes (Figure 1C) and a stronger negative association was found (OR  = 0.44 p = 0.001).

These results point to an at least partially redundant function among the activating genes and they may be all equally involved in PF protection. This hypothesis was corroborated by the regression analyzes, which showed the presence of more than three activating genes as the major factor of differential susceptibility. A similar conclusion was also reported by van der Slik *et al.*, [33] who extensively tested many regression models in type 1 diabetes, showing that activating *KIR* genotypes influence the overall hierarchy of protection or susceptibility. Although the regression analyzes point the presence of more than three genes as the major factor for susceptibility to PF, it is important to remember that this analyze does not consider other KIR receptors. Considering the balance of signals as the possible major responsible for modulating the NK response [32], the ratio activating/inhibitory genes may be the most important factor if all NK receptors could be analyzed together.

A summary of case/control studies of *KIR* and *KIR*+*HLA* combinations in autoimmune diseases are shown in Table 4. In general, the activating *KIR* genes seem to increase the autoimmune response, being regarded as susceptibility factors in several studies. However, the interactions among NK cell receptors and HLA ligands may play a different role in the different diseases.

**Table 4:** Summary of *KIR* association studies regarding autoimmune diseases.

	Present study	^a^Kimoto *et al.*2010 [44] ^b^Pellett *et al.*2007 [45]	^a^Tajik et al. 2001 [46] ^b^Jiao et al.2008 [47]	Garcia-Leon*et al.* 2011 [48]	^a^ Williams *et al.* 2005 [49] ^b^Martin *et al.*2002 [50]	Pellett *et al.* 2007 [45]	Nikitina-Zake*et al.*2004[51]	Yen, *et al.*,2001 [52]	^a^Luszczek *et al.* 2004 [53] ^b^Suzuki *et al.*2004 [54]	Karlsen *et al.*, 2007 [55]
	*Pemphigus foliaceus*	*Systemic lupus erythematosus*	*Ankylosing spondylitis*	*Multiple sclerosis*	*Psoriatic arthritis*	*Scleroderma*	*Diabetes mellitus*	*Rheumatoid vasculitis*	*Psoriasis*	*Sclerosing cholangitis*
*2DL2*							Susceptibility			
*2DL5*	Protection	Protection^a^	Susceptibility^ab^	Susceptibility			Susceptibility		Susceptibility^b^	
*2DS1*	Protection		Susceptibility^a^	Susceptibility	Susceptibility^ab^	Susceptibility	Susceptibility		Susceptibility^ab^	
*2DS1*+*2DS2*− *		Susceptibility^b^				Susceptibility				
*2DS1*+C2			Susceptibility^a^							
*2DS2*					Susceptibility^b^	Susceptibility	Susceptibility	Susceptibility		
*2DS3*	Protection						Susceptibility			
*3DL1*	Susceptibility									
*3DL1*+Bw4			Protection^a^	Protection						
*3DS1*	Protection		Susceptibility^ab^				Susceptibility			
*3DS1*+Bw4	Protection			Susceptibility						
Bw4	Protection		Protection^a^							Protection
Bw4(80I)			Protection^a^							
Bw4(80T)	Protection									
Bw6	Susceptibility									
Haplotype B	Protection								Susceptibility^b^	
HLA-C2										Protection
HLA-Cw03								Susceptibility		
HLA-Cw06									Susceptibility^a^	
More than threeactivating genes	Protection									

The importance of NK cell in autoimmunity mediated by B cell has been studied, [34] showing that NK activation can result in altered antibody responses, including those against autoantigens. Many autoimmune diseases are suspected to be related with infectious etiology, what brings the NK cell, responsible for the first line of defense, to an important role in the protection against autoimmunity. Particularities of PF could explain why the activating KIR function is protecting against this disease. Different from other autoimmune diseases, PF presents extremely high incidence in some endemic geographical areas in Brazil, in sharp contrast to its very low incidence elsewhere in South America and other continents. This unusual characteristic and also the gradual replacement of the endemic area in Brazil beyond years show that one (or more) environmental factor of limited geographic distribution is triggering the disease. Although the exact nature of the environmental contribution is still obscure, there are evidences that a black fly from the Simuliidae family could be involved in the infection with a protein, a virus or a microorganism which could initiate the autoimmune response in genetic susceptible individuals [35]. This agent does not need to result in an overt disease in the exposed individuals but it is related to the onset of pemphigus foliaceus. Moreover, other factors are also associated to PF, such as the low quality of life, the vicinity to rivers, some food, among others [4]; and the patients are usually exposed to this environmental context during their entire life. Many association studies have demonstrated that activating *KIR* genes protect against infectious disease (reviewed by Kulkarni et al., 2008 [25]). Therefore, carrying more activating *KIR* genes could represent a better protection against viruses, microorganisms or other environmental factors that trigger PF. Increasing the number of activating genes means a larger repertory of NK receptors recognizing antigens and activating the NK to defense.

Schleinitz *et al*. [36] presented other hypotheses on the reasons why NK cells could protect against autoimmunity. They could control the presentation of autontigens by killing the immature dendritic cells or control the activation of macrophages, which are responsible for tissue injury in chronic inflammation. However, in this particular disease, the activation of NK cells that control the release of autoantigens, mainly by regulating the environmental factors, such virus or other pathogens, could explain why the activating KIR function may be protective.

Only two inhibitory genes differed between patients and controls. *KIR3DL1* was associated to increased susceptibility (OR  = 2.1; p = 0.03) in the total sample. Because the odds ratios indicated increased susceptibility in both strata (Euro, OR  = 1.93; Afro, OR  = 2.35) we merged the two samples for *KIR3DL1* association analysis This association could be explained by the absence of *KIR3DS1*, once *3DL1* and *3DS1* are alleles of the same locus. The presence of *KIR3DL1* (and consequently the absence of *KIR3DS1*), may generate an increase of inhibitory signals, with reduction of the ratio activating/inhibitory receptors, what could explain this association. On the other hand, the haplotype B gene *KIR2DL5* was negatively associated to PF in Euro-descendants only. We conclude that this association results from the linkage disequilibrium with several activating genes that are associated to PF only in this population stratum. High positive LD values for the pairs *KIR2DL5* and other haplotype B activating genes *(2DS3, 2DS5*) are well recognized and have been reported in many studies, including a Southern Brazilian population of similar ancestry as our study population [20].

The different association patterns between Euro-descendant and Afro-descendant samples could be consequences of the complexity of the disease, allied to differences of other interacting susceptibility factors, whose frequencies may differ between the two population strata. The functional effects of the gene products depend on complex pathways. Other unknown genetic and environmental factors related to PF susceptibility may be involved in complex interactions with the genes here analyzed, and may depend partially on the ethnic context. Among all factors involved in resistance or susceptibility to PF, the activating *KIR* relative effect in Euro-descendants is possibly higher, which means that other unknown factors may predominate in this disease in Afro-descendants.

The frequency of HLA-C1/C2 did not differ between patients and controls, but significant associations were found regarding the Bw4 ligands (Table 3). It is noteworthy that, besides the differential susceptibility to PF being strongly associated with HLA class II genes [6], [7], this is the first study reporting significant association between this disease and the class I genes. The Bw4 epitope was found negatively associated with PF, in agreement with the negative association observed for its cognate activating KIR3DS1 receptor. Therefore, the susceptibility conferred by the presence of Bw6 could be interpreted, in this context, as simple consequence of the absence of Bw4. It is also corroborated by the effect of absence of dominance found between Bw4 and Bw6 epitopes. We cannot discard, however, the possibility of Bw6 acting in a direct susceptibility role. Due its weak binding to its receptor, [18], [19] the stronger association of Bw4(80T) than Bw4(80I) could be an evidence that the KIR binding is not the only reason for this epitope being associated. Although we do not know if *HLA*-*A* and *B* have other implications in PF pathogeneses beyond KIR recognition, a careful search for allelic association of these loci should be performed.

To verify if the association between PF and the HLA Bw4 and Bw6 epitopes could result from LD with the previously associated *HLA-DRB1* alleles, we analyzed the linkage disequilibrium between these class I and class II genes. Interestingly, Bw4(80T), which is significantly less frequent among patients than among controls, was found in negative LD with the protective group of *HLA-DRB1* alleles. The opposite was found for Bw6, which is associated to higher susceptibility to PF and occurs in positive LD with the susceptibility group of *HLA-DRB1* alleles. We cannot affirm that the association found for Bw4 and Bw6 is simply a consequence of the LD with HLA class II alleles or vice versa, but this is possible. The associations of pemphigus foliaceus with Bw4 and with HLA-DRB1 might both result from their functions. Differential epitope selection by the HLA-DRB1 molecules encoded by different alleles is a likely mechanism, but we still do not know if this is the cause of the associations, because of the lack of functional studies. Similarly, it is plausible to hypothesize that the Bw4 epitope has a direct influence on pemphigus foliaceus disease susceptibility, because of its importance for recognition by KIR; however, this remains to be formally demonstrated by functional studies. Notwithstanding, the fact that the association between PF and the combination of *KIR* genes with their cognate HLA ligands is stronger that the associations with the receptors and the ligands individually is an compelling evidence that the interaction of Bw4 by its KIR receptor is directly involved in PF pathogenesis.

Combinations *KIR*+HLA also exhibited significant differences between patients and controls (Figure 2). The presence both *KIR3DS1* and Bw4 showed stronger association than each one separately, which may corroborate that the activating function may be, in fact, the major factor interfering in the PF pathogenesis. Interestingly, the strongest negative association between *KIR*+HLA combinations was found for *KIR3DS1*+HLA-Bw4(80T). There are clear evidences that HLA-Bw4(80I) is powerfully recognized by KIR3DL1, and only indirect evidences that its homologue KIR3DS1 interacts with the same ligand [18], [19], [21]. Nevertheless, the isoleucine at position 80 is not essential for this interaction [37] and the studies performed have regarded mainly KIR3DL1 activity, being the comparisons between the KIR3DS1 ability of distinguishing both Bw4 ligands not well explored. These findings show that Bw4(80T) may also be important as a KIR3DS1 ligand, even if they are less strongly recognized.

*KIR* activating genes, their HLA ligands and combinations of both appear to be protective in the context of PF etiology. This association may be related with the contribution of infectious agents to the pathogenesis of this disease. NK cells have been demonstrated as important for autoreactivity and also have been implicated in several autoimmune diseases. To understand how those cells and their receptors influence the immune response could be a key for the comprehension of one face of the autoimmune complexity.

## Materials and Methods

We analyzed 233 patients and 204 controls without history of the disease. The majority of the patients were contacted at Hospital Adventista do Pênfigo, Campo Grande, MS, Brazil. All individuals voluntarily agreed to participate and provided written informed consent. This study was approved by the Human Research Ethics Committee of the Federal University of Parana, in accordance with the Brazilian Federal laws. The individuals were separated in two groups, according to their predominant ancestry: Euro-descendants (Euro) and Afro-descendants (Afro). The reason of this subdivision is because different populations may differ in allele frequencies. Although this subdivision is arbitrary, this approach was validated by population genetic studies, which showed that the distribution of alleles known to be restricted to populations autochthonous from one continent follows a gradient among the population strata, as expected if the classification discriminated the strata according to relative contributions of the ancestral population [38], [39]. When the frequencies between Euro-descendants and Afro-descendants did not differ statistically, they were analyzed as a single population sample, because the total sample may give us a better representation of the whole population.

The *KIR* genotyping was performed by the PCR-SSP multiplex method [40] using two pairs of primers for each gene. For *KIR2DL2* and *2DL3*, an additional pair of primers for each gene [41] was included to confirm the results. A subset (163 patients and 114 controls) was genotyped for HLA class I applying the LABType® SSO reagent kits (One Lambda, USA).

Frequencies of *KIR* genes and haplogroups were obtained by direct counting. Tests of population differentiation were performed by analyzes of 2×2 contingency tables, calculating the exact p value by the metropolis algorithm. The p value of 0.05 was adopted as the significance limit. The Mantel-Haenszel method [42] applied for calculating the odds ratio (OR) and the 95% confidence intervals. Logistic regression analyzes were carried out by IBM® SPSS® Statistics software. HLA haplotypic frequencies were obtained using the ELB algorithm and the estimates of the LD parameters D′, r^2^, and *p* value were performed using the Arlequin software package [43].

## Supporting Information

Table S1 ***KIR*** **frequencies in patients and controls.** *f* = carrier frequency; *f*G = gene frequency.
(DOC)

Table S2 **Association analyzes for the combinations** ***KIR*** **x HLA ligands.** The significant negative association between pairs *KIR3DL1* and Bw4 epitopes may result from the high frequency of *KIR3DL1* in the population, which means that the association values are explained by the Bw4 epitope frequencies only.
(DOC)
