# Supplementary material for: Activating KIR and HLA Bw4 Ligands Are Associated to Decreased Susceptibility to Pemphigus Foliaceus, an Autoimmune Blistering Skin Disease
Source: PLoS One. 2012 Jul 2;7(7):e39991. doi: 10.1371/journal.pone.0039991 (PMC3388041; doi:10.1371/journal.pone.0039991)
Supplement: Table S1 — KIR frequencies in patients and controls. f = carrier frequency; fG = gene frequency. (DOC) [file pone.0039991.s001.doc]

**Table S1** *KIR* frequencies in patients and controls.

| **Gene** | **Population** | **Patients** | | **Controls** | |
| --- | --- | --- | --- | --- | --- |
|  |  | *f* (%) | *f*G | *f* (%) | *f*G |
| ***KIR2DL1*** | Euro | 97.3 | 0.83 | 97.7 | 0.85 |
|  | Afro | 92.3 | 0.72 | 98.6 | 0.88 |
|  | Total | 95.4 | 0.78 | 98.0 | 0.86 |
| ***KIR2DL2*** | Euro | 52.8 | 0.31 | 53.1 | 0.32 |
|  | Afro | 54.4 | 0.33 | 47.1 | 0.27 |
|  | Total | 53.4 | 0.32 | 51.0 | 0.30 |
| ***KIR2DL3*** | Euro | 88.2 | 0.66 | 82.3 | 0.58 |
|  | Afro | 77.8 | 0.53 | 85.7 | 0.62 |
|  | Total | 84.2 | 0.60 | 83.5 | 0.59 |
| ***KIR2DL4*** | Euro | 100.0 | 1.00 | 100.0 | 1.00 |
|  | Afro | 100.0 | 1.00 | 100.0 | 1.00 |
|  | Total | 100.0 | 1.00 | 100.0 | 1.00 |
| ***KIR2DL5*** | Euro | 45.1 | 0.26 | 62.3 | 0.39 |
|  | Afro | 61.8 | 0.38 | 54.7 | 0.33 |
|  | Total | 59.5 | 0.36 | 59.5 | 0.36 |
| ***KIR2DS1*** | Euro | 31.5 | 0.17 | 49.6 | 0.29 |
|  | Afro | 38.9 | 0.22 | 43.1 | 0.25 |
|  | Total | 34.3 | 0.19 | 47.3 | 0.27 |
| ***KIR2DS2*** | Euro | 49.3 | 0.29 | 53.8 | 0.32 |
|  | Afro | 54.2 | 0.32 | 45.7 | 0.26 |
|  | Total | 51.1 | 0.30 | 51.0 | 0.30 |
| ***KIR2DS3*** | Euro | 18.8 | 0.10 | 33.8 | 0.19 |
|  | Afro | 27.4 | 0.15 | 33.7 | 0.19 |
|  | Total | 21.7 | 0.11 | 33.8 | 0.19 |
| ***KIR2DS4*** | Euro | 95.1 | 0.78 | 94.7 | 0.77 |
|  | Afro | 93.3 | 0.74 | 90.4 | 0.69 |
|  | Total | 94.4 | 0.76 | 93.2 | 0.74 |
| ***KIR2DS5*** | Euro | 28.5 | 0.15 | 36.6 | 0.20 |
|  | Afro | 35.6 | 0.20 | 38.9 | 0.22 |
|  | Total | 31.2 | 0.17 | 37.4 | 0.21 |
| ***KIR3DL1*** | Total | 93.8 | 0.75 | 88.5 | 0.66 |
|  | Afro | 94.4 | 0.76 | 87.7 | 0.65 |
|  | Total | 94.0 | 0.75 | 88.2 | 0.66 |
| ***KIR3DL2*** | Euro | 100.0 | 1.00 | 100.0 | 1.00 |
|  | Afro | 100.0 | 1.00 | 100.0 | 1.00 |
|  | Total | 100.0 | 1.00 | 100.0 | 1.00 |
| ***KIR3DL3*** | Euro | 100.0 | 1.00 | 100.0 | 1.00 |
|  | Afro | 100.0 | 1.00 | 100.0 | 1.00 |
|  | Total | 100.0 | 1.00 | 100.0 | 1.00 |
| ***KIR3DS1*** | Euro | 29.6 | 0.16 | 44.3 | 0.25 |
|  | Afro | 40.4 | 0.23 | 38.4 | 0.21 |
|  | Total | 33.8 | 0.19 | 42.2 | 0.24 |
| ***KIR2DP1*** | Euro | 97.3 | 0.84 | 98.5 | 0.88 |
|  | Afro | 93.3 | 0.74 | 98.6 | 0.88 |
|  | Total | 95.8 | 0.80 | 98.5 | 0.88 |
| ***KIR3DP1*** | Euro | 100.0 | 1.00 | 100.0 | 1.00 |
|  | Afro | 100.0 | 1.00 | 100.0 | 1.00 |
|  | Total | 100.0 | 1.00 | 100.0 | 1.00 |

*f* = carrier frequency; *f*G = gene frequency
