# Supplementary material for: Activating KIR and HLA Bw4 Ligands Are Associated to Decreased Susceptibility to Pemphigus Foliaceus, an Autoimmune Blistering Skin Disease
Source: PLoS One. 2012 Jul 2;7(7):e39991. doi: 10.1371/journal.pone.0039991 (PMC3388041; doi:10.1371/journal.pone.0039991)
Supplement: Table S2 — Association analyzes for the combinations KIR x HLA ligands. The significant negative association between pairs KIR3DL1 and Bw4 epitopes may result from the high frequency of KIR3DL1 in the population, which means that the association values are explained by the Bw4 epitope frequencies only. (DOC) [file pone.0039991.s002.doc]

**Table S2** Association analyzes for the combinations KIR x HLA ligands.

| **KIR + Ligand** |  | **Patients** | | **Controls** | |  |  |  |
| --- | --- | --- | --- | --- | --- | --- | --- | --- |
|  |  | n | F (%) | n | F(%) | P | OR | 95 % CI |
| **3DS1 + Bw4** | Euro | 12 | 11.9 | 26 | 37.7 | <10^-3^ | 0.22 | 0.10 - 0.48 |
|  | Afro | 14 | 22.6 | 13 | 31.7 | 0.300 | 0.62 | 0.25 - 1.52 |
| **3DS1 + Bw4(80I)** | Euro | 12 | 11.9 | 21 | 30.4 | 0.002 | 0.31 | 0.14 - 0.68 |
|  | Afro | 14 | 22.6 | 11 | 26.2 | 0.610 | 0.79 | 0.31 - 1.96 |
| **3DS1 + Bw4(80T)** | Euro | 1 | 1.0 | 9 | 13.4 | 0.001 | 0.07 | 0.008 - 0.54 |
|  | Afro | 2 | 3.3 | 6 | 15.0 | 0.030 | 0.19 | 0.03 - 1.00 |
| **3DL1 + Bw4** | Euro | 62 | 61.4 | 48 | 70.6 | 0.220 | 0.66 | 0.34 - 1.28 |
|  | Afro | 40 | 63.5 | 32 | 78.0 | 0.110 | 0.49 | 0.20 - 1.20 |
| **3DL1 + Bw4(80I)** | Euro | 55 | 54.5 | 40 | 58.8 | 0.837 | 0.84 | 0.45 - 1.56 |
|  | Afro | 36 | 57.1 | 27 | 65.9 | 0.690 | 0.69 | 0.31 - 1.56 |
| **3DL1 + Bw4(80T)** | Euro | 15 | 14.9 | 14 | 20.6 | 0.330 | 0.67 | 0.30 - 1.50 |
|  | Afro | 5 | 7.9 | 12 | 29.3 | 0.004 | 0.21 | 0.07 - 0.65 |

The significant negative association between pairs *KIR3DL1* and Bw4 epitopes may result from the high frequency of *KIR3DL1* in the population, which means that the association values are explained by the Bw4 epitope frequencies only.
